# Evaluation of clinical effects of a multidisciplinary-collaborated cancer support team for gastrointestinal cancer chemotherapy: prospective observational study protocol of M-CAST study

**DOI:** 10.1186/s12876-023-02849-6

**Published:** 2023-06-19

**Authors:** Yohei Iimura, Mitsuko Nakazawa, Yukari Tsuru, Hitomi Togashi, Tomoe Honda, Keisuke Baba, Masaaki Ishibashi, Chieko Sasuga, Naoki Furukawa, Tomoko Sato, Yasuo Matsubara, Ayako Kamisato, Eiko Yoshii, Seiichiro Kuroda, Narikazu Boku

**Affiliations:** 1 Department of Pharmacy, The IMSUT Hospital, The Institute of Medical Science, The University of Tokyo, 4-6-1, Shirokanedai, Minato-Ku, Tokyo, 108-8639 Japan; 2 Department of Nursing, The IMSUT Hospital, The Institute of Medical Science, The University of Tokyo, 4-6-1, Shirokanedai, Minato-Ku, Tokyo, 108-8639 Japan; 3 Department of Clinical Nutrition, The IMSUT Hospital, The Institute of Medical Science, The University of Tokyo, 4-6-1, Shirokanedai, Minato-Ku, Tokyo, 108-8639 Japan; 4 Department of Oncology and General Medicine, The Institute of Medical Science Hospital, The University of Tokyo, 4-6-1, Shirokanedai, Minato-Ku, Tokyo, 108-8639 Japan; 5 Division of Bioethics, Advanced Clinical Research Center, The Institute of Medical Science, The University of Tokyo, 4-6-1, Shirokanedai, Minato-Ku, Tokyo, 108-8639 Japan

**Keywords:** Multidisciplinary-collaborated team, Gastrointestinal cancer, Chemotherapy, Supportive care

## Abstract

**Background:**

Although the multidisciplinary-collaborated team approach in cancer treatment has recently become popular, prospectively evaluated evidence is limited. We started a multidisciplinary-collaborated cancer support team (MCST) to facilitate cooperation across multidisciplinary medical staff in our hospital and established clinical evidence of supportive care. This study aimed to prospectively evaluate the clinical activity and effect of MCST in patients with gastrointestinal cancer receiving chemotherapy.

**Methods:**

This is a single-center, single-arm, observational study. Patients with gastrointestinal cancer scheduled to receive chemotherapy are enrolled and supported by the MCST. The primary endpoints are the number of interventions by medical staff and the number of patients who showed improvement in side effects. The secondary endpoints are the severity of side effects, medical expenses, number of consultations, the acceptance rate of prescription recommendations, adjuvant chemotherapy completion rates, dose intensity, and time required for co-medical intervention. In addition, medical staff and attending physicians evaluate all adverse events.

**Discussion:**

This study is expected to contribute to establishing new cancer-supportive care teams for patients with gastrointestinal cancer receiving chemotherapy and those with cancer receiving chemotherapy.

**Trial registration:**

This trial was registered in the Japan Registry of Clinical Trials (jRCT) as jRCT1030220495.

The date of first registration, 29/11/2022, https://jrct.niph.go.jp/search

**Supplementary Information:**

The online version contains supplementary material available at 10.1186/s12876-023-02849-6.

## Background

Various chemotherapeutic agents that show survival benefits have been available for gastrointestinal cancer in clinical practice since new cytotoxic agents, molecular-targeted agents, and immune checkpoint inhibitors [1–4] have been developed. In addition, based on genome analysis, including liquid biopsy, further progress is expected in treating gastrointestinal cancer [5].

However, various adverse events caused by chemotherapy can deteriorate the patient’s quality of life (QOL). For example, during chemotherapy for gastrointestinal cancer, gastrointestinal toxicities, myelosuppression, neuropathy, skin disorders, and cardiovascular disorders caused by cytotoxic and molecular-targeted agents are frequently observed. In addition, adverse events induced by immune checkpoint inhibitors are occasionally severe and intractable, such as type 1 diabetes, thyroid dysfunction, adrenal dysfunction, pneumonitis, colitis, hepatitis, and cholangitis, among others. Various of these side effects are difficult to manage by the attending physician alone, and uncontrolled adverse events may require dose reduction, cessation, and discontinuation, reducing chemotherapy’s efficacy. Therefore, a multidisciplinary team approach that includes co-medical staff is vital for managing adverse events.

Pharmacists can propose supportive medicine to attending physicians and educate patients on how to use it to reduce symptomatic adverse events and maintain self-adherence to oral anticancer agents. In addition, nurses can propose and educate patients about self-care to prevent and manage adverse events and improve their daily activities. Nutritionists can advise patients on their diet to maintain and improve their nutritional status in cases of nausea and anorexia. However, there have been few reports about support by the multidisciplinary team approach [6–14], and it is recognized that multidisciplinary support teams can improve the patients’ QOL and maximize the therapeutic effects. AMBORA trial [9] suggested that pharmacological/pharmaceutical care significantly reduced severe side effects from oral anticancer drugs compared to those who did not receive such care. Additionally, Marjorie et al. [12] indicated that a pharmacist-led multidisciplinary collaborative approach significantly improved medication adherence to oral anticancer agents. As mentioned above, without the intervention of a multidisciplinary-collaborated team, side effects can be exacerbated, and medication adherence can be reduced. Although no randomized controlled trials of multidisciplinary team interventions exist, the above data suggest that Multidisciplinary-collaborated Cancer Support Team (MCST) improves side effect management.

In our hospital, the Multidisciplinary-collaborated Cancer Support Team (MCST) for supportive care of patients with gastrointestinal receiving chemotherapy was established in 2022 to facilitate a team approach for each patient in cooperation among the team and to establish new evidence of supportive care for sharing the procedure with the staff of our and other hospitals.

However, prospectively evaluated evidence of the multidisciplinary support team approach is limited. Moreover, each multidisciplinary support team procedure should be continuously revised based on the evaluation in a plan-do-check-action (PDCA) cycle to obtain larger effects and more optimal cost-effectiveness, and new activity should be added if necessary.

Therefore, this prospective observational study aimed to evaluate the daily activities of a multidisciplinary support team focusing on patients with gastrointestinal cancer receiving chemotherapy in our hospital. Furthermore, we aimed to present a model case of an MCST approach in cancer treatment by demonstrating the clinical effects of MCST intervention, including improving the QOL of patients.

## Methods

### Study design

This is a single-center prospective observational study. The protocol is performed in accordance with the Declaration of Helsinki and approved by the ethical review board of The Institute of Medical Science, the University of Tokyo (approval number:2022–43-1117). Our standard procedures are approved for clinical use by our hospital and the ethical review board of The Institute of Medical Science, the University of Tokyo (approval number:2022–43-1117). This study was registered in the Japan Registry of Clinical Trials (jRCT) as jRCT1030220495. All patients are required to provide written informed consent.

### MCST

**Fig. 1:**
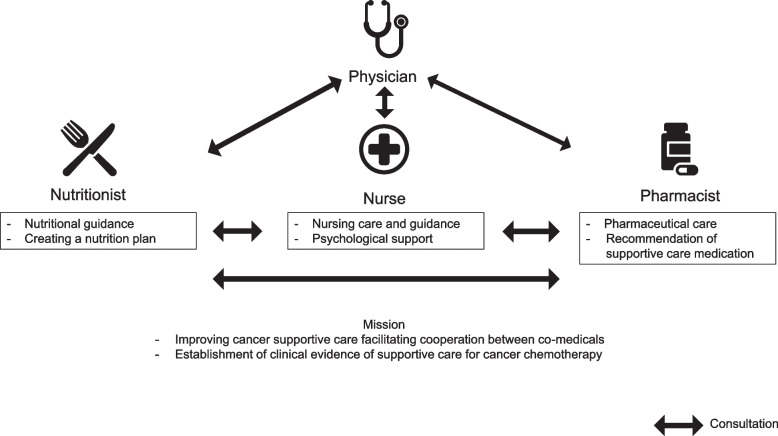
Consultations between co-medicals are permitted in the MCST. With the contract of the cooperation agreement, proposal, patient care, education, and consultation with other medical staff are permitted, if necessary, before the physician’s approval

Two medical oncologists, pharmacists, nurses, and nutritionists, respectively, participated in the MCST. A diagram of the team cooperation is shown in Fig. 1. The MCST aimed to support patients with cancer by providing supportive care in daily practice in cooperation among medical staff and establishing clinical evidence of supportive care for cancer chemotherapy. The following basic roles are pre-specified for each profession: the medical staff evaluated the patients’ condition through interview or telephone before and after the attending physician’s examination. With the contract of the cooperation agreement, proposal, patient care, education, and consultation with other medical staff are permitted, if necessary, before the physician’s approval.

(i) Proposal of medicine for supportive care and modification of chemotherapy regimen by pharmacists
(ii) Instruction of self-care and proposal of social support by nurses
(iii) Nutritional guidance for patients with suspected anorexia and cachexia

### Participants

The inclusion criteria are as follows: (i) patients with gastrointestinal cancer receiving chemotherapy at our hospital; (ii) age ≥ 18 years; and (iii) informed consent to participate in this study. There are no exclusion criteria for this study.

### Chemotherapy

There are two types of chemotherapy, including standard chemotherapy, described in guidelines in Japan, and investigational chemotherapy in clinical trials. The regimen review committee approves all chemotherapy regimens in our hospital, and if the clinical trial protocol allows, both regimens are subject to MCST intervention.

### The procedure of intervention by the MCST

The procedure of each intervention was prespecified ancillary to the protocol. For example, the hand-foot syndrome procedure is included as additional data [see Additional file 1].

### Evaluation

Patient symptoms and laboratory tests will be checked at every visit, and the severity of chemotherapy-induced adverse events will be assessed using the MCST according to the National Cancer Institute Common Terminology Criteria for Adverse Events version 5.0 (CTCAE v5.0).

### Data collection

The following data are collected. Sex, age, type of cancer, stage, prior chemotherapy, chemotherapy regimen, grade of adverse events before and after intervention, intervention and proposal by MCST, the profession of the medical staff performing the intervention, date of intervention, time required for intervention, and medical cost. Data are collected from November 2022 to October 2027.

### Data analysis

Each evaluation item is analyzed using descriptive statistics. According to the severity of side effects before and after the intervention, we calculate the percentage of patients with improved side effects. We calculate the reduction rate of medical expenses before and after the intervention based on the medical expenses during the course before and after the intervention. According to the acceptance rate of the prescription proposal to physicians by the medical staff, the acceptance rate is calculated by dividing the number of accepted recommendations by all recommendations conducted by medical staff to physicians. Furthermore, we evaluate their means based on the adjuvant chemotherapy completion rates, dose intensity, and time required for intervention by medical staff. Therefore, hypothesis testing or *p*-values are not reported in this study.

### Endpoints

The primary endpoints are the number of co-medical interventions and the number of patients improving side effects before and after the intervention. The improvement is determined by the improvement of CTCAE v5.0 rates. The secondary endpoints are (i) the severity of side effects before and after the intervention, (ii) medical expenses during the course before and after the intervention, (iii) the number of consultations between different professions (physician, clinical pharmacists, nurses, and registered dietitians), (iv) acceptance rate of the prescription proposal to physicians by the medical staff, (v) adjuvant chemotherapy completion rates, dose intensity, and (vi) time required for intervention by medical staff.

Most of the side effects can be improved by postponing the treatment or reducing the dose of chemotherapy; however, excessive dose reduction or postponement leads to an inappropriate decrease in dose intensity, which may affect patients’ survival [15–17]. Therefore, it is crucial to maintain the efficacy of chemotherapy while improving patients' QOL by recommending supportive care treatment, appropriate dose reduction, and postponement through MCST intervention. Thus, the endpoint of this study was set as described above.

### Sample size calculation

No statistical sample size calculations were conducted because data analysis in this study are performed by descriptive statistics only, and the confidence interval is not estimable. Our target sample size is 120 patients. The sample size is the number of patients who are expected to receive chemotherapy in our hospital during the study period. We increased the number of cases by an additional 20 to analyze 100 patients assuming a loss to follow-up. Confounding factors were not addressed in this study since no comparative or risk factor analysis is performed. To control for missing data, a margin of 20 cases was provided for the number of cases, as is the case for loss to follow-up.

## Discussion

Recently, pharmacists have been reported to play important roles in supportive care, such as the protocols for cancer-supportive care [6, 11, 18] and the safe use of oral anticancer agents [9, 10]. However, these reports focused on only one profession. Recent studies have shown mixed results regarding team oncology medicine. The activities of multidisciplinary teams started concerning perioperative treatment selection in gastrointestinal cancer treatment [19]. Team approaches, including various professions, such as nurses and social workers, have gradually been reported [7].

To start the team approach, agreement on cooperation based on the specialty and role of each profession is essential. For example, pharmacists assess adverse events and adherence to anti-tumor agents and propose other supportive drugs. In addition, nurses educate and examine the patients’ self-care and introduce the available social resources considering their social background. Nutritionists assess the patient’s nutritional status and daily food content and advise them about the preferable food content. Each role should be well recognized by each medical staff member, and they should collaborate to improve patients’ QOL during chemotherapy. Furthermore, various supports and interventions in cooperation among specialized medical staff are required to resolve the patient’s problem and unmet needs appropriately, and it is preferable that this collaboration can be performed on time by the judgment of each staff member based on the prespecified agreement, even if the attending physicians know retroactively.

As a second step in the team approach, information about the patient’s adverse events and symptoms should be shared. In our MCST, pharmacists examine patients before the attending physician’s examination, hopefully during the waiting time before obtaining the laboratory results. After pharmacists obtain the information, other staff members know additional information. Our MCST activity procedures include methods for sharing information.

This is a master protocol for the prospective evaluation of various activities using MSCT, and each standard procedure is prespecified. However, if the intervention for each adverse event differs among the medical staff, it would be challenging to evaluate. Therefore, we prepared the standard procedures for managing each adverse event in collaboration with the MCST. We believe that this study can accurately evaluate the clinical efficacy of MCST based on standard procedures, including the evaluation of adverse events.

The primary endpoints of this study are the number of co-medical interventions and the number of patients achieving improvement in side effects before and after the intervention. However, dose reduction is the easiest method to reduce adverse events. Therefore, because the dose intensity of anticancer agents can be related to survival [15–17], maintenance of appropriate treatment intensity is necessary for patients, and inadequate dose reduction should be prevented by proposing supportive care. The dose intensity of chemotherapy can be maintained by supporting patients with appropriate side-effect assessments by the medical staff. Therefore, the secondary endpoints of this study are adjuvant chemotherapy completion rates and dose intensity. Moreover, one of the secondary endpoints, which is the time required for each intervention, is also important because of the limited number of medical staff while the number of proposed interventions is increasing. New approaches can be applied to obtain more efficacy by sparing the time required for certain procedures.

**Fig. 2:**
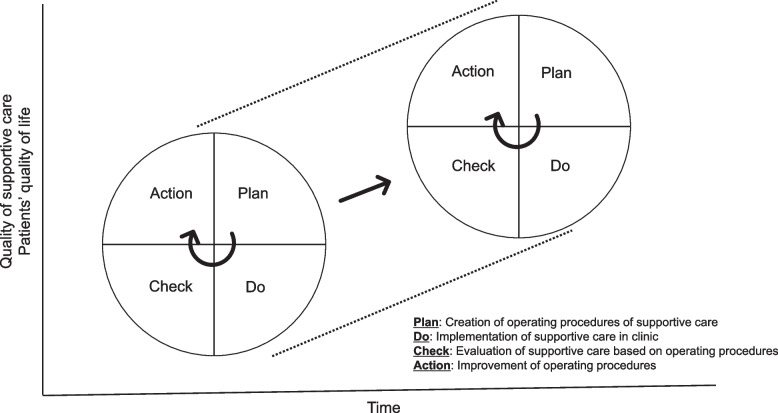
PDCA cycle (plan-do-check-action cycle) is administered and promotes the team's growth centered on improving the operating procedure

This study has some limitations. First, this is a single-center study and not a comparative study. Second, the team approach includes various aspects, making it difficult to determine the most effective intervention. Third, because this study is not a randomized trial, the improvement in outcomes could not be compared. Fourth, because this is a master protocol that covers various interventions, the ancillary procedure of each intervention has not been fully described. However, each prespecified standard procedure and prospective evaluation can improve the activity of the team approach through the PDCA cycle (Fig. 2). This type of prospective study can provide evidence for the care of patients with cancer.

## Supplementary Information

**Additional file 1.** The procedure forhand-foot syndrome induced by chemotherapy.

## Data Availability

The data supporting the findings of this study are available from the corresponding author, YI, upon reasonable request.

## Abbreviations

MCST: Multidisciplinary-collaborated cancer support team
QOL: Quality of life
PDCA: Plan-do-check-action
