# Supplementary material for: Evaluation of clinical effects of a multidisciplinary-collaborated cancer support team for gastrointestinal cancer chemotherapy: prospective observational study protocol of M-CAST study
Source: BMC Gastroenterol. 2023 Jun 19;23:215. doi: 10.1186/s12876-023-02849-6 (PMC10280873; doi:10.1186/s12876-023-02849-6)
Supplement: Supplementary file 1 — Additional file 1. The procedure forhand-foot syndrome induced by chemotherapy. [file 12876_2023_2849_MOESM1_ESM.docx]

The procedure for the hand-foot syndrome (HFS)

1. Purpose

The HFS is a side effect of anticancer chemotherapy that reduces the quality of life (QOL) of patients undergoing chemotherapy. HFS is a skin disorder that appears specifically in the hands and feet, and its main symptoms include numbness, pain, skin redness, keratinization, blisters, and desquamation. Vinorelbine, docetaxel, capecitabine, and molecular target agents, such as regorafenib, sorafenib, and sunitinib, have been reported as causative agents [1-3]. The time of onset of HFS differs depending on the anticancer agent; however, it frequently appears several weeks after the start of chemotherapy, and the symptoms continue with the use of the causative drug. In addition, HFS causes treatment delays, interruptions, and poor medication adherence. Capecitabine is an oral anticancer agent administered for both adjuvant and advanced recurrence chemotherapy in patients with colorectal cancer. As it can be treated on an outpatient basis, it is widely used as a key drug and is recommended by guidelines as a global standard treatment [4]. The incidence of capecitabine-induced HFS ranges from 30% to 80% in all grades of colorectal cancer in randomized controlled trials [5-7]. Management of capecitabine-induced HFS is frequently difficult, and it has been reported that the frequency of occurrence is > 90% in the real world (survey on capecitabine + oxaliplatin combination therapy) [8]. Grade 2 or higher HFS can be associated with swelling, blistering, desquamation, and ulceration, which may cause chemotherapy to be delayed or discontinued. Currently, as supportive care for capecitabine-induced HFS, avoidance of physical stimuli and moisturizing [9-11] are recommended as standard treatments. There are reports examining the effects of topical agents, including exfoliants [12,13], celecoxib [14,15], and oral drugs, such as pyridoxine [16]. However, no high-evidence-level reports exist on the prevention of HFS. Therefore, the only definite improvement in capecitabine-induced HFS is dose reduction and withdrawal [17]. Inhibition of skin basal cell proliferation, drug secretion from eccrine sweat glands, and drug degradation products have been suggested as the mechanisms of HFS development; however, this has not been fully elucidated [18,19] (Table 1). In Table 1, the intervention and control columns are mentioned. The reason for the two columns is to share how effective each intervention is and to be able to make recommendations that suit the situation of symptoms. Another reason is to use the control group value as a reference value when designing new interventions.

Table 1. Summary for clinical evidence of supportive care for HFS

|  |  |  |  |  |  |  |  | **The incidence rate of HFS (any grade)** | | **The incidence rate of HFS (grade2+3)** | | **The incidence rate of HFS (≥grade. 3)** | |
| --- | --- | --- | --- | --- | --- | --- | --- | --- | --- | --- | --- | --- | --- |
|  |  |  |  |  |  | **n** | | **%** | | **%** | | **%** | |
| **Reference No.** | **Study design** | **Intervention** | **Tumor type** | **Capecitabine dose(/times)** | **Observation period** | **Intervention** | **Control** | **Intervention** | **Control** | **Intervention** | **Control** | **Intervention** | **Control** |
| PMID: 21113620 | RCT | Celecoxib | Colorectal cancer | 1250 mg/m2 or 1000 mg/m2 | 3–6  months | 51 | 50 | 29 | 52 | 13.7 | 40 | 1.96 | 10 |
| PMID: 21940785 | RCT | Celecoxib | Colorectal cancer | 1250 mg/m2 or 1000 mg/m2 | 3–6  months | 68 | 71 | 57.4 | 74.6 | 17.6 | 38.1 | 2.9 | 8.5 |
| PMID: 25557587 | RCT | Pyridoxine | GI tract | 973.5 mg/m2 – 1250 mg/m2 | 1 to 3 cycles | 180 | 180 | 76.1 | 64 | 12 | 15.4 | 5.0 | 3.3 |
| PMID: 32406009 | RCT | TJ-28 vs. Pyridoxine | Colorectal cancer | 1250 mg/m2 | N.A. | 12 | 10 | 83.3 | 70.0 | 50 | 40 | 8.3 | 20.0 |
| PMID: 26715292 | Case-control study | Structured teaching program | Colon cancer | 1250 mg/m2 or 1000 mg/m2 | N.A. | 20 | 20 | 52.7 | 50 | 20 | 15.7 | 5.2 | 10 |
| PMID: 32717127 | RCT | Structured Teaching Module | Colorectal cancer | 1000 mg/m2 | 4.5 months  (median) | 140 | 140 | 97.7 | 95.6 | 33.3 | 32.8 | 5.2 | 6.8 |

HFS, hand-foot syndrome; N.A., not available

Based on this background, a multidisciplinary-collaborated cancer support team consisting of physicians, pharmacists, nurses, and nutritionists examined the role of each occupation and treatment guideline and created a supportive care protocol for HFS. This study aimed to prevent HFS and provide early therapeutic intervention to reduce or aggravate HFS, thereby improving the dose intensity of cancer chemotherapy and the patient’s QOL. In addition, we expect that clarification of the role of each occupation will improve the efficiency of work, enhance multidisciplinary team medical care, and improve skills.

1. Role of each occupation

Physicians evaluate HFS, determine whether to continue chemotherapy, prescribe drugs for HFS prevention and treatment, and request dermatology consultation. Nurses evaluate skin disorders in general and provide patients with self-care guidance. Pharmacists evaluate skin disorders, recommend suspension of chemotherapy owing to skin disorders, provide guidance to maintain self-adherence, and propose prescriptions*) for the prevention and management of skin disorders. As it has been suggested that skin disorders are related to nutritional status, nutritionists provide nutritional guidance.

*) If a pharmacist proposes a prescription, it should be within the scope stipulated in the hospital procedure manual, and the pharmacist who recommends the prescription should have more than half a year of outpatient pharmacist assignment experience and be appointed by a Japanese Society of Pharmaceutical Health Care and Sciences-Certified Senior Oncology Pharmacist, which enables prescription recommendations.

1. HFS preventive measures and medical guidance

Before starting chemotherapy, the physicians prescribed the supportive care drugs specified in Table 4. Next, pharmacists explained the dosage and administration of chemotherapeutic agents, efficacy, side effects, and application of topical preparations. In addition, nurses provided instructions on how to cleanse (wash face, bathe/shower), moisturize, and protect (washing, choosing clothes, ultraviolet protection, applying makeup/shaving).

1. Completion of side effect self-report form by the patient

The patient fills in when side effects appear from the date of initiation of the anticancer drug treatment. The completed self-report form will be handed to the medical staff during the outpatient visit and shared with the team.

Table 2. Self-report of adverse events 1

| Side effect self-report 1 | | | | | | | | | |
| --- | --- | --- | --- | --- | --- | --- | --- | --- | --- |
| Name Date | | | | | | | | | |
|  | | Grade |  |  |  |  |  |  |  |
| Stomatitis | I have stomatitis; however, it does not hurt. | 1 |  |  |  |  |  |  |  |
|  | Stomatitis hurts a little; however, I can eat more than half. | 2 |  |  |  |  |  |  |  |
|  | I can hardly eat because of stomatitis. | 3 |  |  |  |  |  |  |  |
| Hand foot syndrome | Red and swollen, but no pain. | 1 |  |  |  |  |  |  |  |
|  | There is pain; however, it does not interfere with daily life. | 2 |  |  |  |  |  |  |  |
|  | It is painful and interferes with daily life. | 3 |  |  |  |  |  |  |  |
| Pigmentation | There is pigmentation in limited, such as fingertips. | 1 |  |  |  |  |  |  |  |
|  | There is systemic pigmentation. | 2 |  |  |  |  |  |  |  |
| Peripheral neuropathy | There is some numbness; however, it does not affect the operation. | 1 |  |  |  |  |  |  |  |
|  | It is difficult to act due to numbness; however, it does not interfere with daily life. | 2 |  |  |  |  |  |  |  |
|  | Numbness interferes with daily life. | 3 |  |  |  |  |  |  |  |
|  | I have no sensation. | 4 |  |  |  |  |  |  |  |
| Peripheral sensory impairment | I react instantly to hypersensitivity when I come in contact with cold things. | 1 |  |  |  |  |  |  |  |
|  | Contact with cold objects sustains a hypersensitive reaction but is painless. | 2 |  |  |  |  |  |  |  |
|  | Contact with cold objects causes persistent hypersensitivity and pain. | 3 |  |  |  |  |  |  |  |
| ＜Please enclose the affected area.＞ | | | | | | | | | |


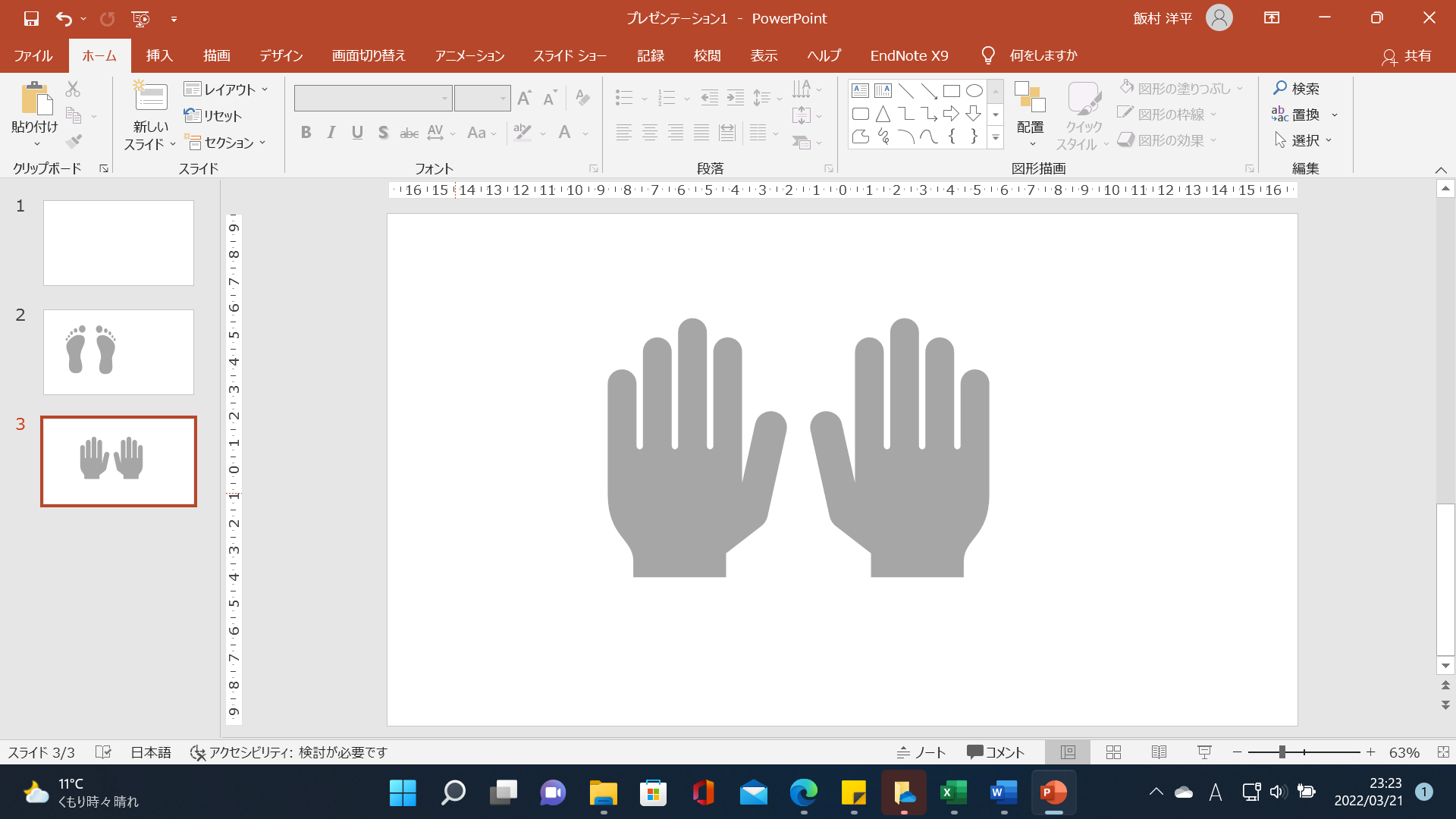

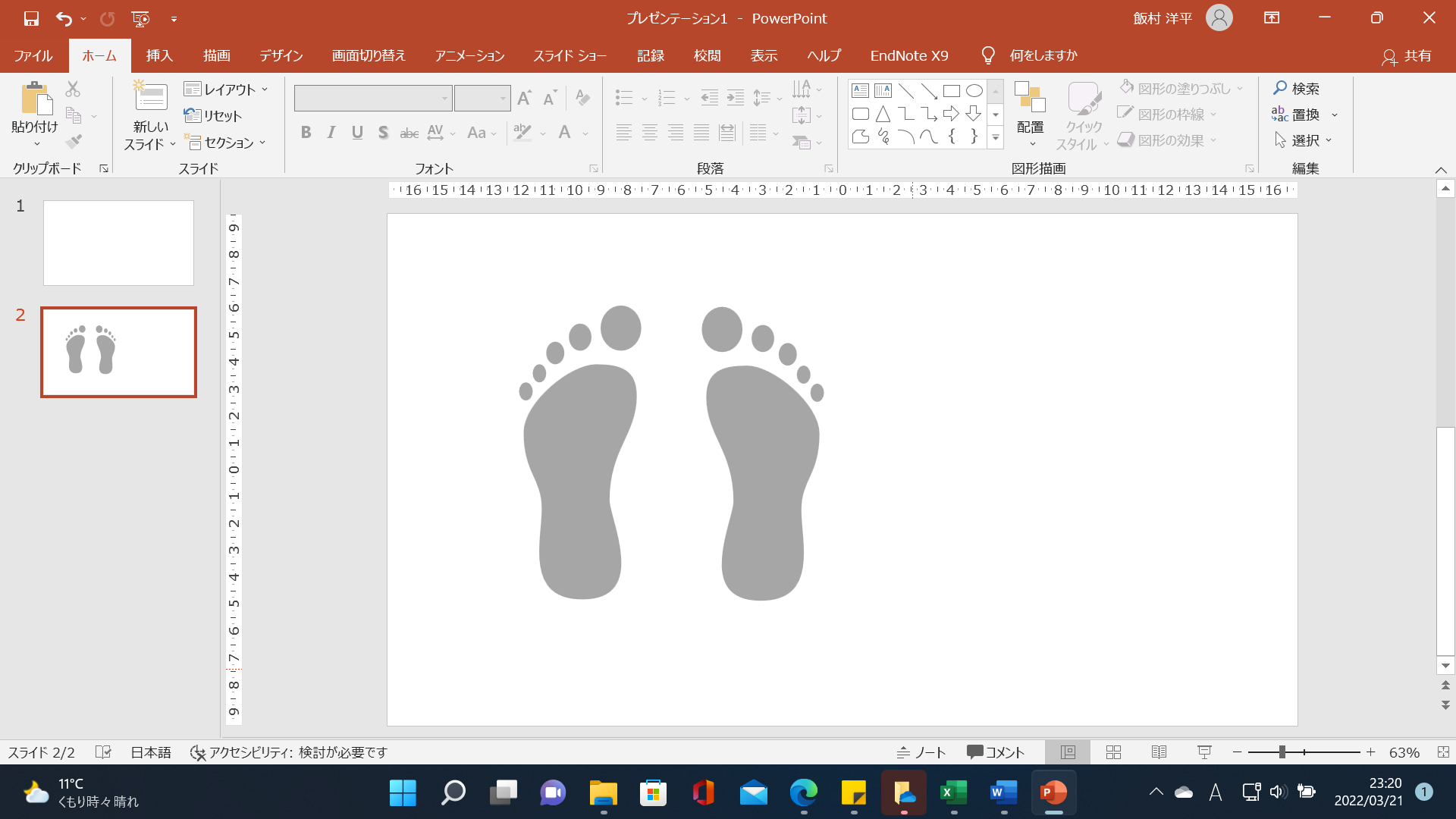


Table 3. Self-report of adverse events 2

| Side effect self-report 2 | | | | | | | | | |
| --- | --- | --- | --- | --- | --- | --- | --- | --- | --- |
| Name Date | | | | | | | | | |
|  | | Grade |  |  |  |  |  |  |  |
| Nausea | Tolerable | 1 |  |  |  |  |  |  |  |
|  | If I use anti-nausea drugs, I can manage to eat. | 2 |  |  |  |  |  |  |  |
|  | I can hardly eat because of nausea. | 3 |  |  |  |  |  |  |  |
| Anorexia | I have a slight loss of appetite. | 1 |  |  |  |  |  |  |  |
|  | I can eat somehow. | 2 |  |  |  |  |  |  |  |
|  | I can hardly eat. | 3 |  |  |  |  |  |  |  |
| Malaise | I am a little tired; however, it does not interfere with my daily life. | 1 |  |  |  |  |  |  |  |
|  | I often lie down. | 2 |  |  |  |  |  |  |  |
|  | I lie down more often than I am awake. | 3 |  |  |  |  |  |  |  |
|  | Bedridden all day long. | 4 |  |  |  |  |  |  |  |
| Diarrhea | Increased defecation frequency less than four times a day compared to usual. | 1 |  |  |  |  |  |  |  |
|  | Increased defecation frequency 4–6 times a day compared to usual. | 2 |  |  |  |  |  |  |  |
|  | Increased defecation frequency more than seven times a day compared to usual. | 3 |  |  |  |  |  |  |  |
| If you have any other symptoms of concern, please write them down. | | | | | | | | | |

1. Evaluation of HFS

Physician evaluation is performed according to CTCAE v5.0.

Pharmacists interviewed the patient before the physicians’ examination, evaluated the grade of the skin disorder based on the interview and the side effect self-report table, and recommended the necessary medicine. In addition, we determined the necessity of chemotherapy suspension according to the grade of the skin disorder and entered the above information in the electronic medical record. Notably, if it is determined during the interview that the method of use of the drug is different from the guidance content or if a new skin disorder countermeasure drug is added, guidance will be provided again. After the pharmacist’s interview, the physicians examined the patient, evaluated the grade of the skin disorder, and confirmed, completed, and ordered supportive care medicine according to recommendations by pharmacists. If prescription modification is necessary for the recommendation of pharmacists, physicians modify the prescription—a comprehensive evaluation of therapeutic and side effects to determine whether chemotherapy should be continued. The nurses assessed the patients’ self-care abilities before the physicians’ visit. Suppose it is determined during the interview that the self-care method differs from the instructions. In that case, re-instructions will be given on the cleaning, moisturizing, protection, and topical agent application methods, and the instructions will be recorded in the electronic medical record. If the grade evaluation of skin disorders differs by occupation, it is not necessary to match; however, the factors should be clarified. If the therapeutic drug is modified after the physician’s examination by physicians or pharmacists if requested.

1. Criteria for withdrawal of chemotherapy

If the patient’s skin disorder progresses to grade 2, the pharmacists will suggest chemotherapy withdrawal to the physicians. During the examination, physicians comprehensively evaluated the effects and side effects and determined the treatment policy.

1. Supportive care medicine

In principle, the medicine should be selected from Table 4; however, the change in dosage form should be approved according to the patient’s wishes or the medical staff’s judgment. If it is determined that medications other than those listed below are necessary, consult physicians separately.

Table 4. Prescription recommendation for HFS

| Severity | Prevention | Grade 1 | Grade 2 | Grade 3 |
| --- | --- | --- | --- | --- |
| External agent | 1. Heparinoid 2. Topical   hydrocortisone (medium class steroid)*) | 1. Medium class steroid | 1. Very strong class steroid**) | 1. Strongest class steroid 2. Nadifloxacin If with infection |
| Consultation with a dermatologist |  |  |  | consideration |

*) If obtained an informed consent to participate in T-CRACC study [20].

**) Regarding the class of topical steroids, even if symptoms are grade 2, using the strongest class should be considered depending on the site and symptoms.

HFS, hand-foot syndrome

1. Reference

1. Nagore E, Insa A, Sanmartín O. Antineoplastic therapy-induced palmar plantar erythrodysesthesia (‘hand-foot’) syndrome. Incidence, recognition and management. Am J Clin Dermatol. 2000;1:225-34.

2. Grothey A, Van Cutsem E, Sobrero A, Siena S, Falcone A, Ychou M, et al. Regorafenib monotherapy for previously treated metastatic colorectal cancer (CORRECT): an international, multicentre, randomised, placebo-controlled, phase 3 trial. Lancet. 2013;381:303-12.

3. Nardone B, Hensley JR, Kulik L, West DP, Mulcahy M, Rademaker A, et al. The effect of hand-foot skin reaction associated with the multikinase inhibitors sorafenib and sunitinib on health-related quality of life. J Drugs Dermatol. 2012;11:e61–5.

4. NCCN Guidelines® for Colon Cancer, version 2.2021. <https://www.nccn.org/patients/guidelines/content/PDF/colon-patient.pdf>. Accessed January 10, 2023.

5. Grothey A, Sobrero AF, Shields AF, Yoshino T, Paul J, Taieb J, et al. Duration of adjuvant chemotherapy for Stage III colon cancer. N Engl J Med. 2018;378:1177-88.

6. Yamazaki K, Yamanaka T, Shiozawa M, Manaka D, Kotaka M, Gamoh M, et al. Oxaliplatin-based adjuvant chemotherapy duration (3 versus 6 months) for high-risk stage II colon cancer: the randomized phase III ACHIEVE-2 trial. Ann Oncol. 2021;32:77-84.

7. Hamaguchi T, Shimada Y, Mizusawa J, Kinugasa Y, Kanemitsu Y, Ohue M, et al. Capecitabine versus S-1 as adjuvant chemotherapy for patients with stage III colorectal cancer (JCOG0910): an open-label, non-inferiority, randomised, phase 3, multicentre trial. Lancet Gastroenterol Hepatol. 2018;3:47-56.

8. Yokokawa T, Kawakami K, Mae Y, Sugita K, Watanabe H, Suzuki K, et al. Risk factors exacerbating hand-foot skin reaction induced by capecitabine plus oxaliplatin with or without bevacizumab therapy. Ann Pharmacother. 2015;49:1120-4.

9. Son HS, Lee WY, Lee WS, Yun SH, Chun HK. Compliance and effective management of the hand-foot syndrome in colon cancer patients receiving capecitabine as adjuvant chemotherapy. Yonsei Med J. 2009;50:796-802.

10. Van Cutsem E, Twelves C, Cassidy J, Allman D, Bajetta E, Boyer M, et al. Oral capecitabine compared with intravenous fluorouracil plus leucovorin in patients with metastatic colorectal cancer: results of a large phase III study. J Clin Oncol. 2001;19:4097-106.

11. Blum JL, Jones SE, Buzdar AU, LoRusso PM, Kuter I, Vogel C, et al. Multicenter phase II study of capecitabine in paclitaxel-refractory metastatic breast cancer. J Clin Oncol. 1999;17:485-93.

12. Lu W, Huang Z, Chen S, Lv H, Chen X, Lei J, et al. The effectiveness of EVOSKIN®Palm and sole moisturizing cream in treating capecitabine-associated hand-foot syndrome: a randomized double-blind clinical trial. Ann Palliat Med. 2021;10:3009-17.

13. Wolf SL, Qin R, Menon SP, Rowland KM Jr, Thomas S, Delaune R, et al. Placebo-controlled trial to determine the effectiveness of a urea/lactic acid-based topical keratolytic agent for prevention of capecitabine-induced hand-foot syndrome: North Central Cancer Treatment Group Study N05C5. J Clin Oncol. 2010;28:5182-7.

14. Zhang RX, Wu XJ, Lu SX, Pan ZZ, Wan DS, Chen G. The effect of COX-2 inhibitor on capecitabine-induced hand-foot syndrome in patients with stage II/III colorectal cancer: a phase II randomized prospective study. J Cancer Res Clin Oncol. 2011;137:953-7.

15. Zhang RX, Wu XJ, Wan DS, Lu ZH, Kong LH, Pan ZZ, et al. Celecoxib can prevent capecitabine-related hand-foot syndrome in stage II and III colorectal cancer patients: result of a single-center, prospective randomized phase III trial. Ann Oncol. 2012;23:1348-53.

16. Jo SJ, Shin H, Jo S, Kwon O, Myung SK. Prophylactic and therapeutic efficacy of pyridoxine supplements in the management of hand-foot syndrome during chemotherapy: a meta-analysis. Clin Exp Dermatol. 2015;40:260-70.

17. Scheithauer W, Blum J. Coming to grips with hand-foot syndrome. Insights from clinical trials evaluating capecitabine. Oncology (Williston Park). 2004;18:1161-8, 1173; discussion 1173-6, 1181-4.

18. Diasio RB. Oral DPD-inhibitory fluoropyrimidine drugs. Oncology (Williston Park). 2000;14;Suppl 9:19-23.

19. Degen A, Alter M, Schenck F, Satzger I, Völker B, Kapp A, et al. The hand-foot-syndrome associated with medical tumor therapy - classification and management. J Dtsch Dermatol Ges. 2010;8:652-61.

20. Iimura Y, Furukawa N, Ishibashi M, Ahiko Y, Tanabe T, Aikou S, et al. Study protocol of a single-arm phase 2 study evaluating the preventive effect of topical hydrocortisone for capecitabine-induced hand-foot syndrome in colorectal cancer patients receiving adjuvant chemotherapy with capecitabine plus oxaliplatin (T-CRACC study). BMC Gastroenterol. 2022;22:341.

Additional file 1 – The procedure for hand-foot syndrome induced by chemotherapy
